# Reply to Yan et al.: Quercetin possesses a fluorescence quenching effect but is a weak inhibitor against SARS-CoV-2 main protease

**DOI:** 10.1073/pnas.2309870120

**Published:** 2023-09-05

**Authors:** Haiyu Xu, Yute Zhong, Jinyue Yang, Lifeng Fu, Yi Shi, Luqi Huang, George F. Gao

**Affiliations:** ^a^Institute of Chinese Materia Medica, Academy of Chinese Medical Sciences, Beijing 100700, China; ^b^Chinese Academy of Sciences Key Laboratory of Pathogen Microbiology and Immunology, Institute of Microbiology, Chinese Academy of Sciences, Beijing 100101, China; ^c^State Key Laboratory of Dao-di Herbs, National Resource Center for Chinese Materia Medica, China Academy of Chinese Medical Sciences, Beijing 100700, China

Recently, we published an article (1) showing that several bioactive compounds were identified from Huashi Baidu decoction (Q-14) that showed both antiviral activity and anti-inflammatory effects against COVID-19. The main protease (M^pro^) of SARS-CoV-2 is an attractive target for the development of antiviral drugs. Our results showed that quercetin and echinatin from Q-14 displayed moderate inhibition activities against SARS-CoV-2 M^pro^ at submicromolar levels by the fluorescence resonance energy transfer (FRET)–based protease activity assay. However, Yan et al. suggested that quercetin is a promiscuous M^pro^ inhibitor because of its fluorescence quenching effect (2).

**Fig. 1.**
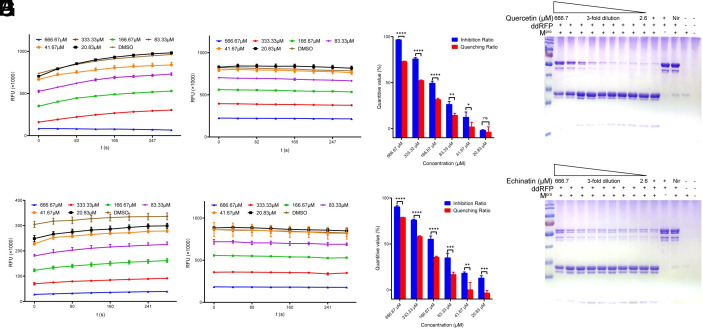
SARS-CoV-2 Mpro inhibitory activity of quercetin and echinatin. (*A*) Inhibition of Mpro by quercetin by the FRET assay. (*B*) The fluorescence quenching effect of quercetin on MCA-AVLQ fragment. (*C*) The Mpro inhibition ratio and quenching ratio of quercetin were calculated (*P < 0.05, **P < 0.01, ***P < 0.001, ****P < 0.0001, and ns: not significant). (*D*) Inhibition of Mpro cleavage of ddRFP by quercetin based on the SDS-PAGE assay. Nirmatrelvir (Nir) was used as a positive control. (*E*) Inhibition of Mpro by echinatin by the FRET assay. (*F*) The fluorescence quenching effect of echinatin on MCA-AVLQ fragment. (*G*) The Mpro inhibition ratio and quenching ratio of echinatin were calculated (*P < 0.05, **P < 0.01, ***P < 0.001, ****P < 0.0001, and ns: not significant). (*H*) Inhibition of Mpro cleavage of ddRFP by echinatin based on the SDS-PAGE assay. Nirmatrelvir (Nir) was used as a positive control.

We took this suspicion very seriously. Therefore, we repeated the FRET-based protease activity assay and tested the fluorescence quenching effects with the MCA-AVLQ-Lys (Dnp)-Lys-NH2 (MCA-AVLQ) fragment. The FRET assay results showed that both quercetin and echinatin have M^pro^ inhibition activity (Fig. 1 *A* and *E*), but both quercetin and echinatin displayed a fluorescence quenching effect on MCA-AVLQ fragment (Fig. 1 *B* and *F*). The inhibition ratio and quenching ratio were further calculated (Fig. 1 *C* and *G*). We found that the inhibition ratio is significantly greater than the quenching rate, especially at low concentrations, which indicates that quercetin and echinatin should possess certain M^pro^ inhibition activity. To further validate the M^pro^ inhibition activity of these two compounds, we performed an Sodium dodecyl sulfate – polyacrylamide gel electrophoresis (SDS-PAGE) assay of the cleavage of the Dimerization-dependent red fluorescent protein (ddRFP) biosensor, and this method can avoid the interference of fluorescence quenching effects which was also used by Yan et al. (3). We found that quercetin has obvious M^pro^ inhibition activity at high concentrations (from 166.67 μM to 666.67 μM), using nirmatrelvir (PF-07321332) as a positive control. By contrast, echinatin showed minor M^pro^ inhibition activity at 666.67 μM (Fig. 1 *D* and *H*).

In conclusion, we confirmed that quercetin and echinatin showed weak M^pro^ inhibition activity, although fluorescence quenching effects were observed by Yan et al. (2) and by us here. We cannot rule out the possibility that these two compounds might have other targets to achieve the anti-SARS-CoV-2 effects, which can be further studied in the future. We agree that we should be cautious with our assay methods, and it is necessary to verify the inhibitory activity by different methods due to the limitations of current available methods.
